# A Strategic Implementation Framework for Integrating Pharmacogenomics (PGx) into Personalized Healthcare in Saudi Arabia

**DOI:** 10.3390/genes17091115

**Published:** 2026-09-14

**Authors:** Kholud R. Alrubaie, Raghad A. Alzaylaee, Latifah A. Alayyaf, Farjah H. Algahtani, Shaker A. Alomary, Abdullah M. Assiri, Mariam M. AlEissa

**Affiliations:** 1 Population Health Observatory, Ministry of Health, Riyadh 11176, Saudi Arabia; 2 Oncology Center, Chair of Epidemiology and Public Health Research, King Saud University, Riyadh 12373, Saudi Arabia; falgahtani@ksu.edu.sa; 3 College of Medicine, Alfaisal University, Riyadh 11533, Saudi Arabia; 4 Research Department, King Khaled Eye Specialist Hospital, Riyadh 11462, Saudi Arabia; 5 Public Health Laboratory, Public Health Authority, Riyadh 11176, Saudi Arabia; 6 KAPSARC School of Public Policies (KSPP), King Abdullah Petroleum Studies and Research Center (KAPSARC), Riyadh 11672, Saudi Arabia

**Keywords:** pharmacogenomics, population health observatory, Saudi Arabia, precision medicine, clinical implementation, personalized medicine, Vision 2030

## Abstract

Pharmacogenomics has the potential to improve the safety and efficacy of medicines in Saudi Arabia, and has a strong basis for implementation through population-specific genomic evidence, growing laboratory capacity and national digital health investment. We undertook a structured policy analysis and evidence synthesis of Saudi, regional, and international evidence; compared selected implementation capabilities across established models; and assessed national readiness descriptively across key health system domains. Using published evidence and structured expert judgement, we propose gene–drug priority tiers according to their clinical impact, population relevance and feasibility. Saudi Arabia’s strengths are in genomic evidence and digital health infrastructure, but large gaps exist in governance, laboratory standardization, electronic health record interoperability, clinical decision support, workforce readiness, reimbursement, and outcome evaluation. Five pathways are proposed for initial implementation: *CYP2C19*–clopidogrel, *DPYD*–fluoropyrimidines, *TPMT*/*NUDT15*–thiopurines, HLA-guided anticonvulsant prescribing and HLA-B*57:01–abacavir. We propose a framework that could be supported by the Population Health Observatory and a six-phase roadmap, moving from governance and technical design to multicenter pilots, national scale-up and continuous evaluation.

## 1. Introduction

Pharmacogenomics (PGx) is the science of how inherited genetic variation influences medication response, treatment efficacy, toxicity, and dose requirements. It can support safer drug selection and more precise dosing [1,2]. Its clinical implementation, however, depends on translating genetic results into clear prescribing actions at the point of care. Evidence-based guidelines from the Clinical Pharmacogenetics Implementation Consortium (CPIC) and the Dutch Pharmacogenetics Working Group (DPWG) explain how available genotype results should inform drug selection and dosing, but they do not by themselves define when testing should be ordered or how services should be delivered [3,4]. Guidelines must therefore be supported by validated laboratories, standardized interpretation, electronic health record integration, clinical decision support, reimbursement, data governance, and a trained workforce [2,5].

Saudi Arabia has several foundations that may support the national implementation of PGx. These foundations indicate potential relevance and capacity, but do not by themselves establish clinical utility, testing demand, or health system readiness. The Kingdom is investing in healthcare transformation, digital infrastructure, and data-driven care [6]. In a study of 11,889 Saudi individuals, 99.2% were found to carry at least one actionable variant in eight pharmacogenes [7]. Analyses of Saudi exome and human leukocyte antigen data have also identified clinically relevant and population-specific variation [8]. Health system prescribing data have revealed substantial exposure to medicines influenced by pharmacogenes [9]. A tertiary care pilot in Riyadh further demonstrated that PGx testing can be integrated into electronic records and clinical workflows [10].

Important implementation barriers remain. Saudi healthcare professionals report gaps in PGx knowledge and confidence [11], and regional evidence identifies limitations in education, infrastructure, regulation, and reimbursement [12]. International implementation evidence, including the PREPARE study, shows that genotype-guided prescribing can reduce clinically relevant adverse drug reactions when testing is connected to prescribing guidance and clinical workflows [13]. The Saudi evidence supports the clinical relevance of several gene–drug pathways [14], but successful scale-up still requires coordinated governance, laboratory quality, digital integration, financing, and continuous evaluation [15].

PGx is a practical component of precision medicine [16,17]. Its international implementation has progressed from isolated reactive testing toward guideline-driven and pre-emptive models that connect reusable genotype results with electronic health records and point-of-care decision support [18,19]. In Saudi Arabia, this direction aligns with Vision 2030 and the Health Sector Transformation Program, which promote integrated, value-based, and digitally enabled healthcare [20]. The development of a high-quality Saudi reference genome further supports population-specific implementation, although local validation and coordinated national standards remain necessary [21]. In this paper, the PHO is treated as having a proposed national public health intelligence function rather than as an established national PGx infrastructure. This policy paper therefore aims to assess Saudi readiness, identify implementation gaps, prioritize high-impact gene–drug pathways, and propose a PHO-enabled national framework and phased implementation roadmap.

### What This Study Adds

This study contributes to the pharmacogenomics implementation literature by integrating population-specific genomic evidence, health system readiness, clinical prioritization, digital interoperability, governance, financing, workforce development, and implementation outcomes into a single national framework. Rather than thinking of pharmacogenomic testing as a stand-alone lab intervention, we think of PGx as a population health capability dependent on coordinated translation of genomic information into prescribing decisions and measurable clinical outcomes.

The proposed framework is characterized by three contributions. We first synthesize a multidimensional national PGx readiness assessment, evaluating the scientific, regulatory, laboratory, digital, workforce, financial and outcome-evaluation requirements for implementation. Second, we propose a transparent, multi-criteria rationale for prioritizing gene–drug pathways based on clinical impact, population relevance, preventability, and feasibility. Third, we conceptualize the Population Health Observatory as a national monitoring and learning layer that can link genomic evidence to prescribing, pharmacovigilance, outcomes and equity indicators, while maintaining prescribing decisions within clinical care systems. The framework is not only for Saudi Arabia but is also a transferable model for other countries with emerging genomic infrastructure.

## 2. Methods

### 2.1. Study Design and Objectives

This study used a structured policy analysis and evidence synthesis design to assess readiness for national PGx implementation in Saudi Arabia and to develop a prioritized implementation framework. The analysis examined clinical actionability, population relevance, governance, laboratory capacity, electronic health record interoperability, clinical decision support, workforce readiness, financing, data governance, ethics, and implementation outcomes.

### 2.2. Search Strategy and Source Selection

We utilized open sources like PubMed/MEDLINE, Google Scholar, clinical guideline repositories, and relevant governmental and professional sources, and searched for Saudi, regional, and international evidence. The searches were started on 22 January 2026 and last updated on 29 July 2026. Because the review was not designed for exhaustive study identification, no formal source identification count was generated; 56 sources were included in the final synthesis. The complete search strategy, information sources, search dates, eligibility criteria, and source types are presented in Supplementary Table S1. The search concepts included pharmacogenomics, pharmacogenomics, Saudi Arabia, clinical implementation, CPIC, DPWG, electronic health records, clinical decision support, governance, reimbursement, and precision medicine. The study team conducted source identification, eligibility assessment, and evidence synthesis, purposively selecting 56 relevant sources that covered at least one prespecified implementation domain. The appraisal was conducted collectively by the study team through an integrated discussion, with differences in interpretation resolved by consensus; no independent duplicate scoring was performed. The scores represent descriptive, evidence-informed capability ratings rather than validated quantitative maturity measures; the supporting evidence, rationale, and uncertainty for each domain are presented in Supplementary Table S2.

### 2.3. Evidence Appraisal and Analysis

The evidence was categorized by source type. The MMAT domains, AGREE II principles, and the RE-AIM dimensions of reach, effectiveness, adoption, implementation, and maintenance were used as informal conceptual guides for examining empirical studies, clinical guidelines, and implementation programs, respectively; no formal appraisal scores were generated. The evidence review and analysis were undertaken by the study team; no separate independent scoring procedure was applied because the tools were used conceptually. International models were compared across areas of governance, testing strategy, laboratory organization, EHR and clinical decision support integration, workforce preparation, financing, portability of results, and outcome evaluation. Because the benchmarked entities represented different system types, the comparisons were limited to selected capabilities and were not interpreted as overall national PGx maturity. Saudi readiness was assessed across eight domains: evidence base, clinical adoption, workforce readiness, digital infrastructure, financing, outcome evaluation, laboratory capacity, and governance and ethics. The readiness scores were descriptive capability ratings: 0, no identified capability; 1, emerging or limited capability; 2, partially established capability; and 3, operationalized capability. The ratings were based on structured expert judgement of the reviewed evidence and were not validated quantitative maturity scores. Each readiness score was assigned collectively by the study team based on the available published evidence and the predefined operational definitions; areas of uncertainty were conservatively assigned a lower score, and differences in interpretation were resolved through consensus without independent duplicate scoring. The gene–drug pathways were prioritized according to their expected clinical and population health impact and implementation feasibility, as informed by the evidence strength, Saudi population relevance, test availability, laboratory complexity, turnaround requirements, clarity of prescribing action, and workflow integration. The criteria were not numerically weighted. Tier 1 comprised the pathways considered high impact and high feasibility; Tier 2 comprised the pathways considered high impact but requiring greater testing, monitoring, or workflow complexity. The assignments were evidence-informed expert judgements rather than outputs of a validated instrument.

The Saudi-specific inputs included the population variation, prescribing exposure, tertiary care implementation, oncology practice, and adverse drug reaction evidence described in Section 1 and Section 4.1 [7,8,9,10,14,22,23].For each pathway, the available Saudi evidence on allele frequency, medication exposure, clinical burden, and testing capacity was considered; where pathway-specific national data were unavailable, this uncertainty was explicitly documented in the prioritization findings and considered when assigning the proposed priority tier.

### 2.4. Evidence Synthesis and Planned Outputs

The evidence was narratively integrated to generate five linked outputs: an assessment of evidence quality, an international benchmark comparison, a Saudi readiness and gap assessment, a prioritized list of gene–drug pathways, and a PHO-enabled national framework with a phased implementation roadmap. No quantitative meta-analysis was conducted. Because the review was designed for policy development rather than for exhaustive study identification, it was treated as a structured policy analysis rather than a formal systematic review.

## 3. Results

### 3.1. Evidence Quality and Implementation Assessment

The reviewed evidence varied in its design, strength, and applicability. The Saudi evidence was dominated by observational, cross-sectional, and single-institution studies, whereas international implementation programs provided more consistent evidence on feasibility, adoption, and EHR/clinical decision support integration. Guideline recommendations were generally strongest regarding the clarity of genotype-based prescribing actions, while applicability, reimbursement, local adaptation, long-term maintenance, equity, and economic value varied across settings.

The established guidelines and international implementation experience were therefore considered appropriate foundations for the proposed model. However, Saudi clinical effectiveness, cost-effectiveness, sustainability, and regional equity were identified as priorities for prospective evaluation during multicenter pilot implementation. The diverse international strategies for pharmacogenomics implementation are summarized in Table 1.

### 3.2. International Benchmark Findings

The following comparative matrix outlines key international best-practice frameworks for pharmacogenomics (PGx) implementation, detailing core components, clinical evidence, and strategic implications for the Saudi healthcare context (Table 1).

### 3.3. Saudi Readiness and Gap Assessment

**Figure 1:**
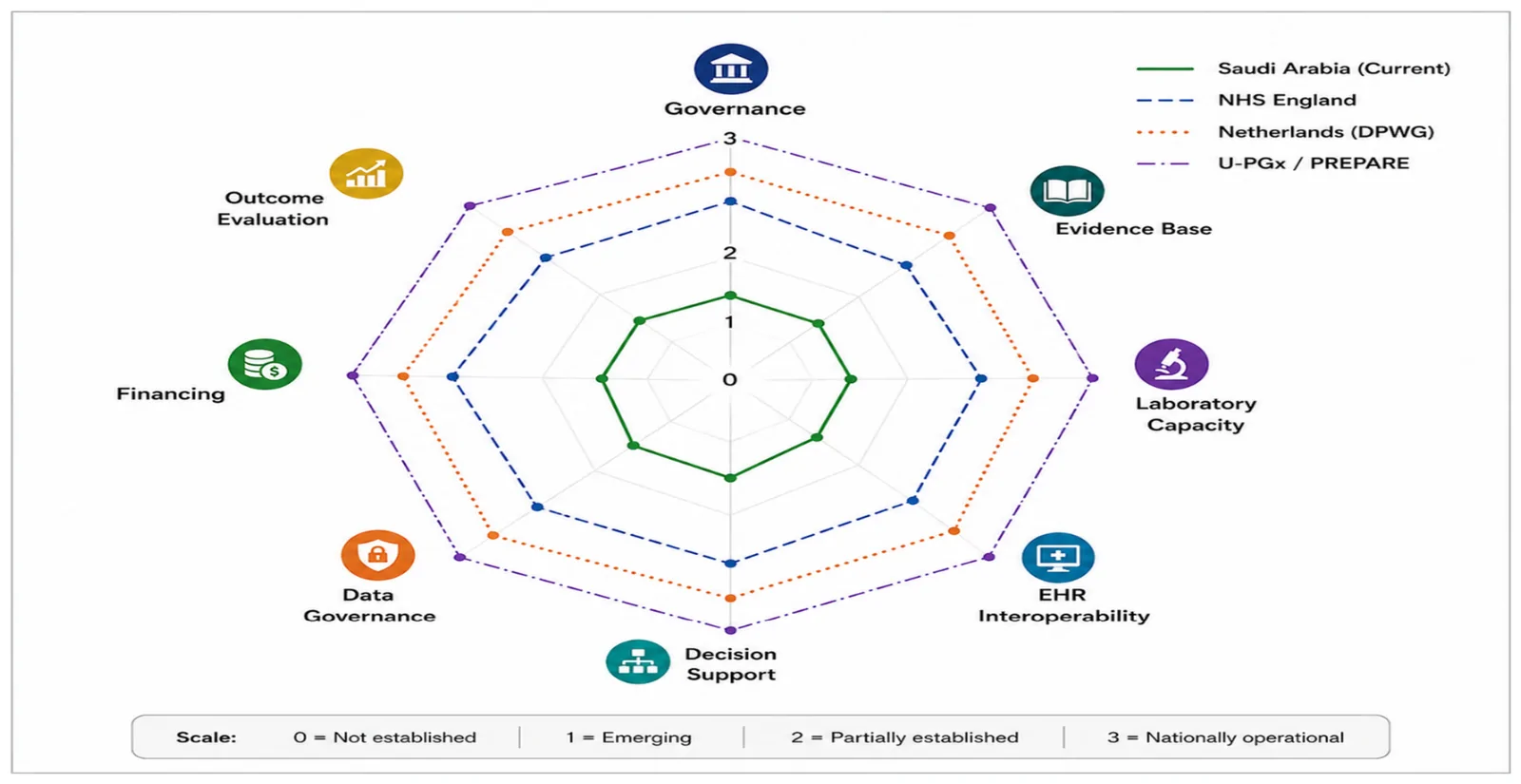
Illustrative comparison of selected PGx implementation capabilities across heterogeneous systems using 0–3 descriptive scale. Scores represent evidence-informed capability ratings rather than validated measures of overall national PGx maturity. Supporting evidence, rationale, and uncertainty are presented in Supplementary Table S2. Created by authors.

The readiness assessment identified substantial differences between the current Saudi PGx environment and mature international implementation systems. Eight domains were considered: evidence base, clinical adoption, workforce readiness, digital infrastructure, financing, outcome evaluation, laboratory capacity, and governance and ethics. Figure 1 provides an illustrative comparison of selected capabilities across heterogeneous systems; it does not represent a comparison of overall national PGx maturity. Because the benchmarked entities represent different functions and levels of healthcare infrastructure, Figure 1 provides an illustrative comparison of selected implementation capabilities and should not be interpreted as a comparison of overall national PGx maturity.

Saudi strengths include increasing population-specific genomic evidence, national investment in digital health transformation, emerging molecular laboratory capacity, and early institutional experience with precision medicine. These findings indicate that PGx is technically feasible in selected tertiary care settings, although the current capabilities are unevenly distributed and are not yet connected through standardized national prescribing pathways.

The principal gaps involve governance, interoperability, clinical workflow integration, and laboratory standardization. National testing indications, common genotype-to-phenotype translation rules, structured reporting requirements, and clearly assigned clinical responsibilities have not yet been established. PGx results are not consistently stored as portable, reusable EHR data, and real-time decision support is not routinely available when an included medicine is prescribed. Laboratory access, assay content, reporting formats, and turnaround times also vary across institutions.

Additional limitations are identified in workforce preparation, financing, and evaluation. Published surveys indicate gaps in professional knowledge, confidence, and practical training, while reimbursement pathways for testing, interpretation, and follow-up remain undefined. Saudi evidence on clinical effectiveness, cost-effectiveness, sustainability, and regional equity is also limited. Overall, Saudi Arabia has a credible scientific and technical foundation for PGx, but national implementation requires coordinated governance, laboratory quality assurance, interoperable EHR and clinical decision support infrastructure, workforce development, sustainable financing, and continuous outcome monitoring.

### 3.4. Gene–Drug Prioritization Findings

**Figure 2:**
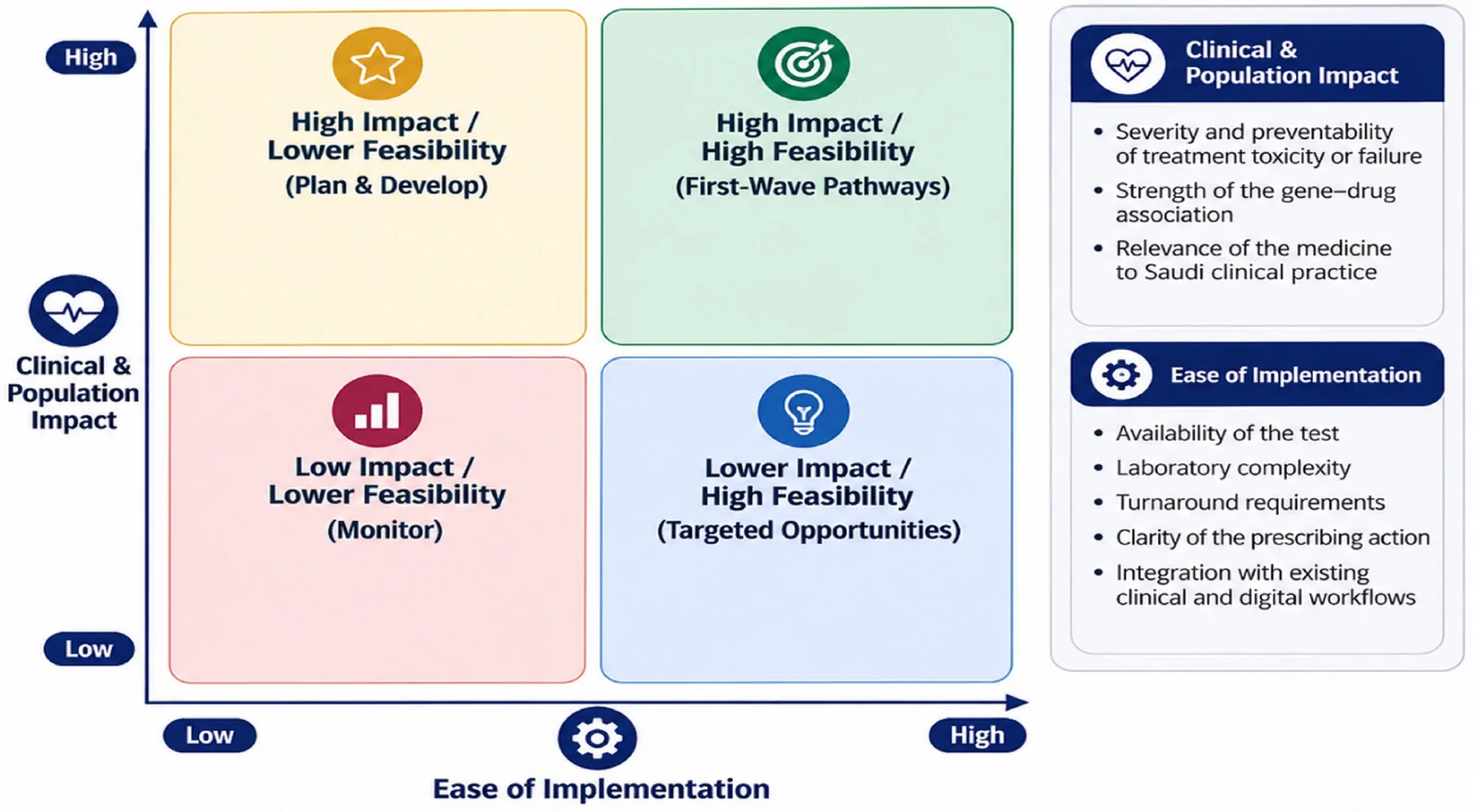
Conceptual priority matrix for gene–drug pairs. Created by the authors.

The conceptual priority matrix (Figure 2) organizes pathway assessment according to two axes: expected clinical and population health impacts, and ease of implementation in the Saudi healthcare system. The clinical impact is based on the severity and preventability of treatment toxicity or failure, the strength of the gene–drug association and the expected relevance of the medicine to Saudi clinical practice. Feasibility is driven by the availability of the test, laboratory complexity, turnaround requirements, clarity of the prescribing action and the ability to integrate the pathway into existing clinical and digital workflows. Figure 2 does not display the individual pathway placements; the proposed assignments are reported in Table 2.

The evidence-informed prioritization proposed five high-impact, high-feasibility pathways for first-wave implementation: *CYP2C19*–clopidogrel, *DPYD*–fluoropyrimidines, *TPMT*/*NUDT15*–thiopurines, HLA-guided anticonvulsant prescribing and HLA-B*57:01–abacavir. These pathways were chosen because they target serious preventable toxicity or treatment failure and provide specific prescribing instructions [29,30].

Warfarin, tacrolimus and allopurinol were targeted for early expansion because they require more complex testing, rapid turnaround, therapeutic monitoring or locally validated algorithms. In particular, clinical factors and continuous INR monitoring should be incorporated with genotype-guided warfarin dosing [14,31,32]. Additional priority gene–drug pathways, their clinical implications and recommended actions are summarized in Table 2.

**Table 2:** Proposed priority gene–drug pathways for phased pharmacogenomics implementation in Saudi Arabia.

Gene or Biomarker	Drug or Drug Class	Clinical Implication	Recommended Clinical Action	Saudi-Specific Evidence and Remaining Uncertainty	Proposed Priority Tier in Saudi Arabia
*CYP2C19*	Clopidogrel	Intermediate and poor metabolizers have reduced formation of the active metabolite, weaker platelet inhibition and an increased risk of ischemic events, particularly after acute coronary syndrome or percutaneous coronary intervention.	Consider prasugrel or ticagrelor instead of clopidogrel in intermediate and poor metabolizers when not contraindicated [29].	Saudi population-level pharmacogenetic variation and exposure to PGx-relevant medicines have been reported [7,8,9], and local tertiary care implementation has been demonstrated [10]; however, pathway-specific national allele frequency, utilization, and outcome data remain limited.	Tier 1—first wave: High clinical impact in acute coronary syndrome/PCI, clear prescribing action, and feasible targeted testing.
*DPYD*	Fluorouracil and capecitabine	Reduced or absent *DPD* activity increases the risk of severe or potentially fatal gastrointestinal, hematological, neurological and cardiac toxicity.	Reduce the starting dose in intermediate metabolizers and titrate according to tolerance. Avoid fluoropyrimidines in poor metabolizers when an effective alternative is available [33].	Saudi oncology practice shows heterogeneous implementation: approximately 20% of surveyed oncology specialists routinely screen for DPD deficiency, whereas 41% have never requested relevant molecular testing [22]. National allele frequency and testing capacity data remain limited.	Tier 1—first wave: Severe preventable toxicity, clear dose modification, and existing but heterogeneous Saudi oncology testing experience [22].
*TPMT* and *NUDT15*	Mercaptopurine, thioguanine and azathioprine	Reduced or absent *TPMT* or *NUDT15* function increases active thioguanine nucleotide exposure and the risk of severe thiopurine toxicity, particularly myelosuppression.	Reduce the initial dose in intermediate metabolizers. Consider an alternative or a substantially reduced regimen in poor metabolizers with hematological monitoring [34].	General Saudi pharmacogenetic variation supports potential relevance [7,8]; however, pathway-specific Saudi allele frequency, thiopurine utilization, toxicity burden, and testing capacity data are unavailable or insufficient.	Tier 1—first wave: Severe myelosuppression risk, clear phenotype-dependent dose modification, and feasible targeted testing.
HLA-B*15:02 and HLA-A*31:01	Carbamazepine; HLA-B*15:02 also informs oxcarbazepine use	HLA-B*15:02 is associated primarily with carbamazepine- and oxcarbazepine-induced Stevens–Johnson syndrome and toxic epidermal necrolysis, whereas HLA-A*31:01 is associated with carbamazepine-induced maculopapular exanthema drug reaction with eosinophilia and systemic symptoms, Stevens–Johnson syndrome and toxic epidermal necrolysis.	Avoid carbamazepine in treatment-naive carriers when an appropriate alternative is available. Avoid oxcarbazepine in HLA-B*15:02-positive patients [35].	Saudi HLA variation and exposure to pharmacogenetically relevant medicines have been reported [8,9], but pathway-specific national allele frequency, anticonvulsant utilization, and testing capacity data remain insufficient.	Tier 1—first wave: Severe preventable toxicity and clear avoidance action; local allele frequency and medication utilization remain uncertain.
HLA-B*57:01	Abacavir	Carriers have a substantially increased risk of abacavir hypersensitivity, which may become life-threatening after re-exposure.	Do not initiate or reintroduce abacavir in HLA-B*57:01-positive patients. Record the result as a permanent contraindication [30].	Saudi HLA variation has been characterized [8]; however, national HLA-B*57:01 frequency, abacavir utilization, testing uptake, and population-level clinical burden data remain insufficient.	Tier 1—first wave: Life-threatening hypersensitivity and a clear contraindication; local allele frequency and medication utilization remain uncertain.
*CYP2C9*, *VKORC1* and *CYP4F2*	Warfarin	Genetic variation contributes to differences in dose requirements and the risk of excessive or inadequate anticoagulation.	Use a validated pharmacogenetic algorithm incorporating genetic and clinical variables. Continue INR monitoring and dose adjustment [31].	Saudi pharmacogenetic evidence supports potential clinical relevance [7,8,14], but national prescribing exposure, algorithm validation, testing capacity, and prospective outcome data remain limited.	Tier 2—early targeted expansion: High clinical relevance but greater algorithmic complexity and continued INR monitoring requirements.
*CYP3A5*	Tacrolimus	*CYP3A5* expressers generally require higher initial doses to achieve therapeutic concentrations.	Consider a higher genotype-guided starting dose in expressers while maintaining therapeutic drug monitoring [36].	Local PGx services have expanded into transplant care [37,38]; however, Saudi-specific *CYP3A5* frequency, tacrolimus utilization, genotype-guided dosing outcomes, and testing capacity data remain incompletely characterized.	Tier 2—specialist implementation: Specialist use requiring genotype-guided initial dosing together with therapeutic drug monitoring.
HLA-B*58:01	Allopurinol	Carriers have an increased risk of severe cutaneous adverse reactions, including Stevens–Johnson syndrome and toxic epidermal necrolysis.	Avoid allopurinol in positive patients and select an appropriate alternative urate-lowering therapy [32].	Saudi HLA evidence supports potential population relevance [8], but national HLA-B*58:01 frequency, allopurinol exposure, severe cutaneous adverse reaction burden, and testing capacity data remain limited.	Tier 2—targeted expansion: Severe preventable toxicity and clear avoidance action, with local allele frequency and testing capacity requiring validation.

### 3.5. Proposed National PGx Framework and PHO-Enabled Architecture

**Figure 3:**
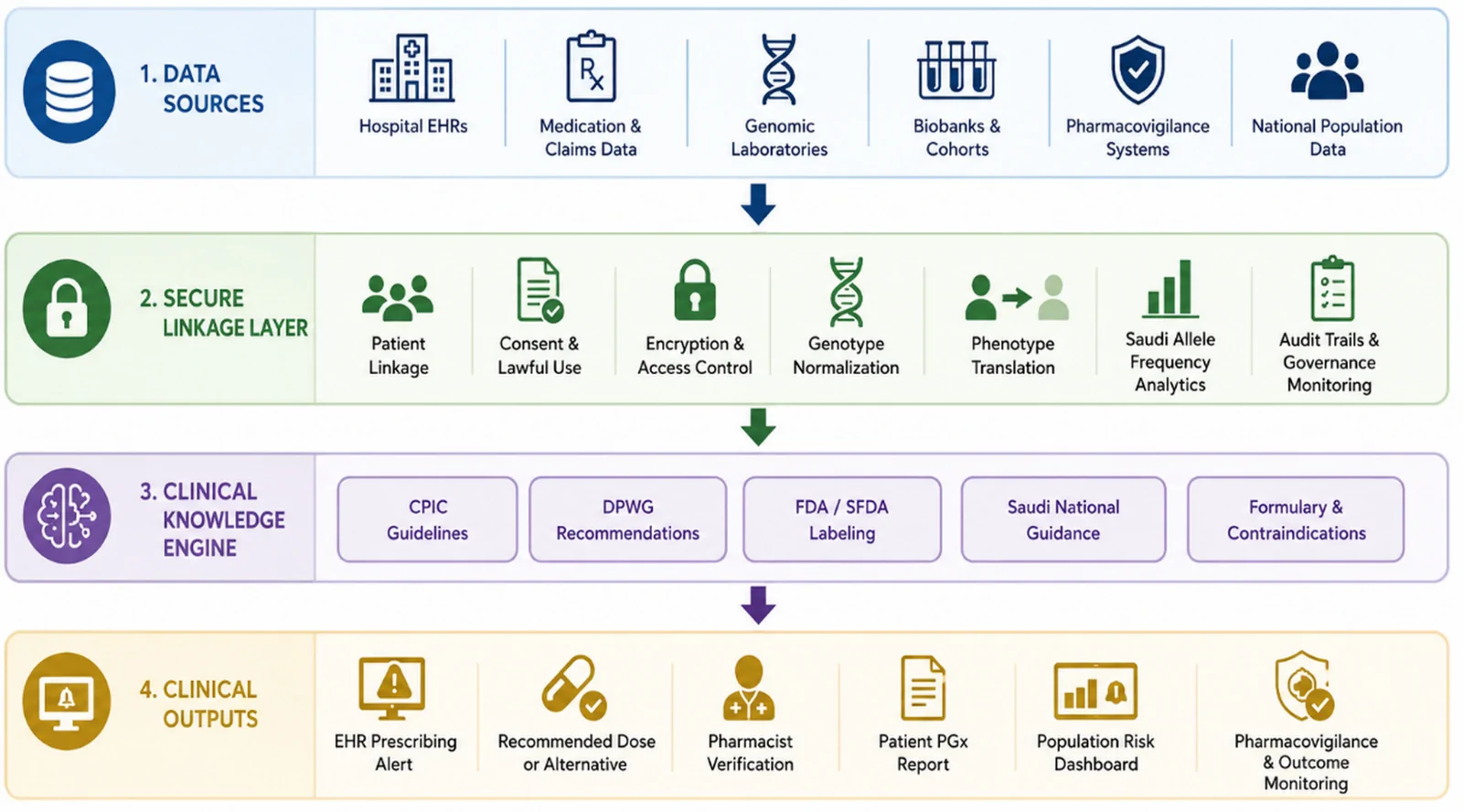
Prioritized national PGx framework and PHO-enabled architecture. Created by the authors.

The prioritization and readiness findings are translated into a nationally governed, locally delivered PGx framework supported by the Population Health Observatory (PHO) architecture (Figure 3). The PHO functions as a secure linkage, standardization, analytics, and monitoring layer rather than replacing hospital EHRs, laboratory systems, or clinical prescribing platforms. The framework connects national governance and clinical standards with accredited testing, interoperable data, point-of-care decision support, and continuous outcome evaluation. The PHO is presented as a proposed national public health intelligence structure, not an established national PGx infrastructure. Equivalent linkage, standardization, analytics, and monitoring functions could alternatively be assigned to another nationally mandated governance architecture.

#### 3.5.1. Data Sources and National Governance

The architecture begins with six main data sources: hospital EHRs, medication and claims data, genomic laboratories, biobanks and cohorts, pharmacovigilance systems and national population health data. A national multidisciplinary PGx committee should define approved testing indications, minimum laboratory standards, genotype-to-phenotype rules and clinical responsibilities. It should also maintain the national priority list and update recommendations when new Saudi or international evidence becomes available.

The data should be shared in standardized, computable formats. NPHIES currently provides health information and financial interoperability functions; its implementation guide does not provide the additional clinical genomic interoperability required for PGx [39]. The HL7 Clinical Genomics Implementation Guide provides a more specific architecture for the exchange of variants, genotypes, interpretations and genomic reports and could guide the development of a Saudi PGx interoperability profile [40].

#### 3.5.2. PHO Secure Linking and Standardization Layer

The PHO linkage layer needs to provide accurate patient matching, consent or other lawful use documentation, encryption, role-based access control and full audit trails. Genomic and clinical data are sensitive personal data and must be processed according to the Saudi Personal Data Protection Law and its implementing requirements [41]. The governance model should also be in line with international principles of privacy, non-discrimination, transparency and fair distribution of the benefits of genomic data [42].

In this secure layer, laboratory results must be standardized, with genotype normalization and phenotype translation. The Saudi allele frequency analytics could then be used to assess local panel coverage, identify under-represented variants and support future refinement of national testing priorities. Individual prescribing decisions should be limited to the authorized clinical systems, and the PHO should mainly facilitate governed linkage, population surveillance and policy evaluation. This function is consistent with the broader mandate proposed for a national Population Health Observatory, which is designed to link and standardize health data streams to strengthen public health intelligence and policy evaluation in Saudi Arabia [43].

#### 3.5.3. Laboratory and Testing Network

The framework should be supported by accredited regional laboratories coordinated through a national reference laboratory. Assay validation, allele coverage, quality control, turnaround time, reporting and external quality assessment all require common specifications. ISO 15189:2022 provides internationally accepted requirements for quality and competence in medical laboratories and should inform the national accreditation pathway [44].

Two complementary testing paths are needed: first, rapid targeted testing should be available to support urgent or drug-triggered decisions, while validated multigene panels should support specialty-based and later pre-emptive testing. A Saudi tertiary care pilot has already shown that PGx testing can be incorporated into an EHR-supported clinical workflow, with 512 patients tested via cardiology and neurology pathways, providing local proof of operational feasibility [10].

#### 3.5.4. Clinical Knowledge Engine

The clinical knowledge engine should include CPIC guidelines, DPWG recommendations, FDA and SFDA labeling, Saudi national guidance, formulary restrictions and patient-specific contraindications. The CPIC and DPWG give a therapeutic interpretation to an available genotype, while the proposed Saudi guidance should also define when testing is indicated and how it should be delivered locally [4,18,29].

Each recommendation should be version-controlled, associated with a defined genotype or phenotype, drug, clinical context and prescribing action. Structured results should lead to an appropriate dose recommendation, alternative medicine, additional monitoring requirement or contraindication. Clinical decision support being integrated into the EHR is a key mechanism for translating PGx information into routine care, rather than having results included in non-computable laboratory reports [45].

#### 3.5.5. Clinical Outcomes and Service Delivery

The framework should provide six key clinical outputs: an EHR prescribing alert, a recommended dose or alternative, pharmacist verification, a patient-facing PGx report, population-level dashboards and pharmacovigilance and outcome monitoring. Clinical pharmacists should assist with interpretation, medication reconciliation, prescriber consultation, and patient counseling during early implementation.

The patient report should be available for future prescribing and across care settings. For a regionally relevant example, the emirate-wide health information exchange in Abu Dhabi, Malaffi, provides PGx reports to eligible clinicians [28]. It shows how genomic reports can be portable from provider to provider, rather than staying locked in the institution that created the test.

#### 3.5.6. Workforce, Financing, and Continuous Evaluation

Implementation will require role-specific training for prescribers, clinical pharmacists, laboratory specialists, nurses and informatics teams. Reimbursement should cover testing, interpretation and clinical follow-up, not just the laboratory procedure. National standards should also cover consent, patient access to results, cybersecurity, and equitable availability across regions.

Preferably, de-identified or pseudonymized data feeds should be securely transmitted to the PHO for national monitoring. Indicators should include testing uptake, turnaround time, actionable results, modification of prescribing, CDS acceptance and overrides, adverse drug reactions, treatment failure, hospitalization, cost and regional access. These data would provide a continuous feedback loop through which the national committee could revise testing panels, clinical rules and priorities for implementation. The learning system approach is supported by the PREPARE study, which showed that pre-emptive panel testing linked to prescribing guidance can reduce clinically relevant adverse drug reactions [13].

#### 3.5.7. The Role of the Population Health Observatory in Sustaining PGx Implementation

The Population Health Observatory (PHO) is proposed here as having a wider national public health intelligence function rather than as a disease- or program-specific unit, and pharmacogenomics is one of a number of areas where its mandate for linkage, standardization and analytics could be deployed [43]. In this role, the PHO would not generate clinical recommendations itself, but instead would aggregate de-identified signals from hospital EHRs, genomic laboratories, biobanks and cohorts, medication and claims data, and pharmacovigilance systems (Section 3.5.1) to support the national PGx committee in reviewing testing uptake, prescribing changes, adverse event patterns and regional access, in the same way that observatory-style intelligence has been proposed to strengthen population health monitoring and outcomes more broadly in Saudi Arabia [46].

This role would be similar to the current Saudi experience of central coordination of population-level genetic and screening programs, while clinical decisions would be left to the treating teams. Nationally coordinated neural tube defect and newborn screening programs illustrate the capacity of a centrally governed program to define testing indications, standardize laboratory pathways, and monitor outcomes across institutions [47,48]. The emerging Saudi biobanking infrastructure offers similar potential for centrally organized, ethically governed reuse of genomic and clinical data for research and quality improvement [49]. The PHO applied to PGx could help the implementation framework by connecting the outputs of national monitoring with the clinical knowledge engine (Section 3.5.4) and enabling the iterative refinement of PGx implementation as Saudi real-world evidence becomes available.

### 3.6. National Implementation Roadmap

**Figure 4:**
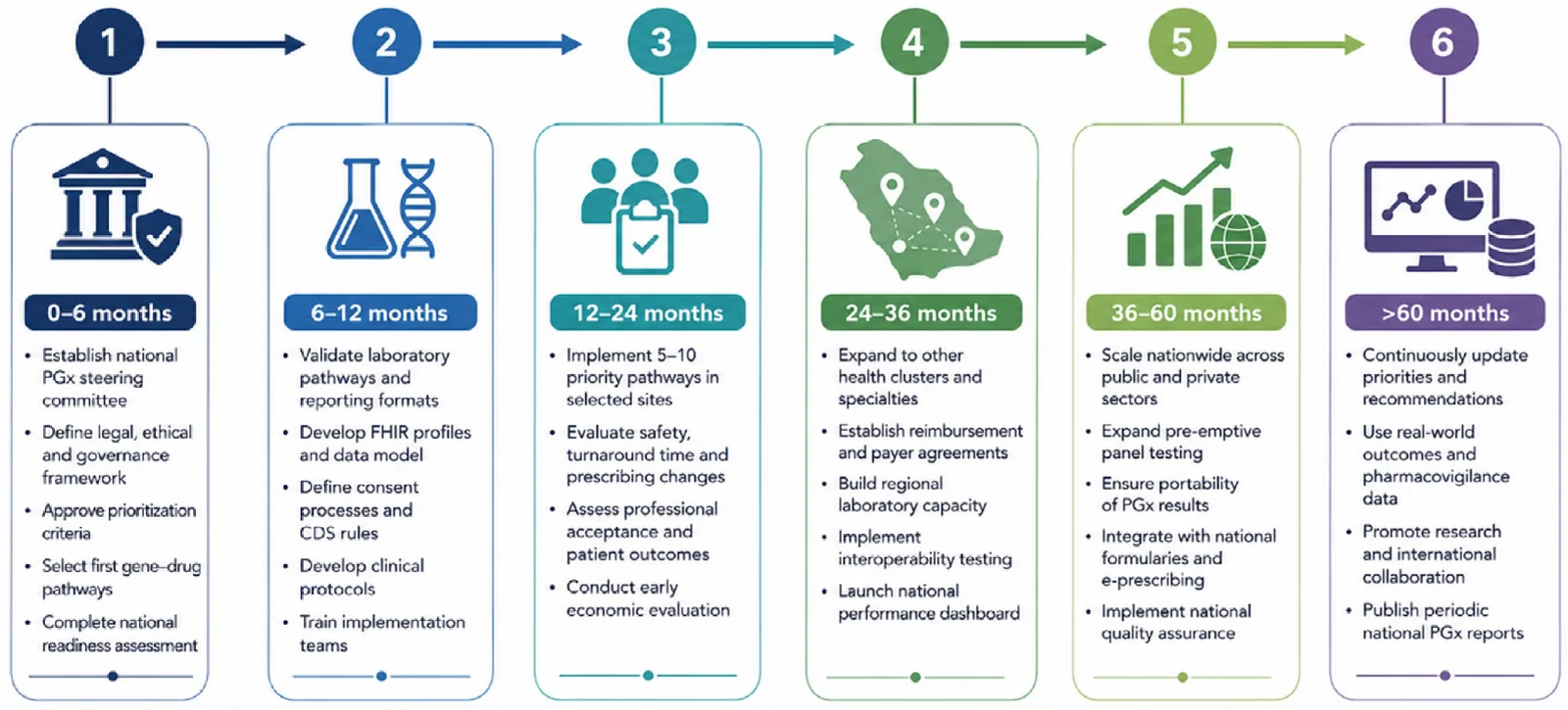
National implementation roadmap. Created by the authors.

The proposed roadmap (Figure 4) operationalizes the prioritized PGx framework into six sequential implementation phases. Each phase has clear activities and deliverables. Progression should be based on demonstrated readiness, safety and performance, not time alone.

**Phase 1: Governance and evidence base (0–6 months)**

A national PGx steering committee should be formed with supporting legal, ethical and technical working groups. This phase should validate the prioritization criteria, approve the initial gene–drug pathways, and complete a national readiness assessment. The key outputs should include a governance charter, ethical framework, national priority list, baseline assessment and criteria for the selection of pilot sites.

**Phase 2: Technical and clinical design (6–12 months)**

In the second phase, the technical and clinical requirements for implementation should be defined, including validated laboratory pathways, reporting formats, FHIR-compatible data profiles, consent processes, clinical protocols, and CDS rules. The initial implementation teams should receive training, and the digital architecture should be tested before clinical go-live. The outputs should include laboratory specifications, a structured PGx data model, validated alerts, clinical protocols, and a workforce training package.

**Phase 3: Multisite pilot (12–24 months)**

Five to ten priority pathways should be implemented in selected healthcare institutions. Their evaluation should include safety, turnaround time, availability of results, prescribing changes, professional acceptance, patient outcomes, and early economic effects. The main deliverables should be a pilot evaluation report, refined clinical and digital rules, an initial economic analysis, and a decision on progression to wider implementation.

**Phase 4: Regional expansion (24–36 months)**

Successful pathways should be extended to additional health clusters and clinical specialties. Regional laboratory capacity, reimbursement mechanisms, and interoperability testing should be established, together with a national performance dashboard comparing implementation across sites. The key deliverables should include operational regional PGx services, payer agreements, interoperability results, and comparative performance reports.

**Phase 5: National scale-up (36–60 months)**

Validated pathways should be implemented across public and private healthcare sectors. The PGx results should be portable across institutions and integrated with national formularies and prescribing systems. Expanded pre-emptive panel testing could be considered for selected populations with high exposure to PGx-relevant medicines. The expected outputs include a nationally coordinated PGx service, portable patient results, expanded formulary integration, and national quality assurance processes.

**Phase 6: Learning health system (beyond 60 months)**

The final stage should ensure regular updating of testing priorities and prescribing recommendations based on Saudi real-world outcomes, pharmacovigilance data, prescribing patterns, and genomic evidence. International collaboration should provide an exchange of knowledge and be consistent with Saudi legal and governance requirements. The expected outputs include a continuously updated national PGx knowledge engine, a research platform linked to outcomes and periodic national PGx reports.

## 4. Discussion

The findings show that national PGx implementation could be constrained by interconnected governance, laboratory, digital, workforce, financial, ethical, and evidentiary barriers. Each barrier should therefore be linked to an accountable policy response and a measurable implementation indicator.

### 4.1. Emerging Pharmacogenomics Implementation in Saudi Arabia

Pharmacogenomics implementation in Saudi Arabia is transitioning from research-based to clinical application; however, development is still confined to a few settings. PGx testing was initially used in cardiology and neurology at King Faisal Specialist Hospital and Research Centre, but it has been utilized in more than 1500 patients and expanded to organ transplant services with a dedicated pharmacist-led PGx clinic [37,38]. Cancer care has also shown emerging activity. A national survey of Saudi oncology specialists revealed that 20% of them routinely screened for DPD deficiency before fluoropyrimidine therapy, while 41% never ordered a relevant molecular test, indicating early but heterogeneous uptake across institutions [22]. Recently, Saudi researchers used 2035 real-world reports of adverse drug reactions or therapeutic inefficacy involving 450 medicines to prioritize drugs for future clinical PGx research [23]. Collectively, these developments indicate increasing clinical and research interest, but published evidence of standardized implementation outside specialized centers is limited. Hence, national mapping of active clinical, pilot and research programs is needed to document gene–drug pathways, testing volumes, prescribing changes, outcomes, digital integration and regional access.

**Figure 5:**
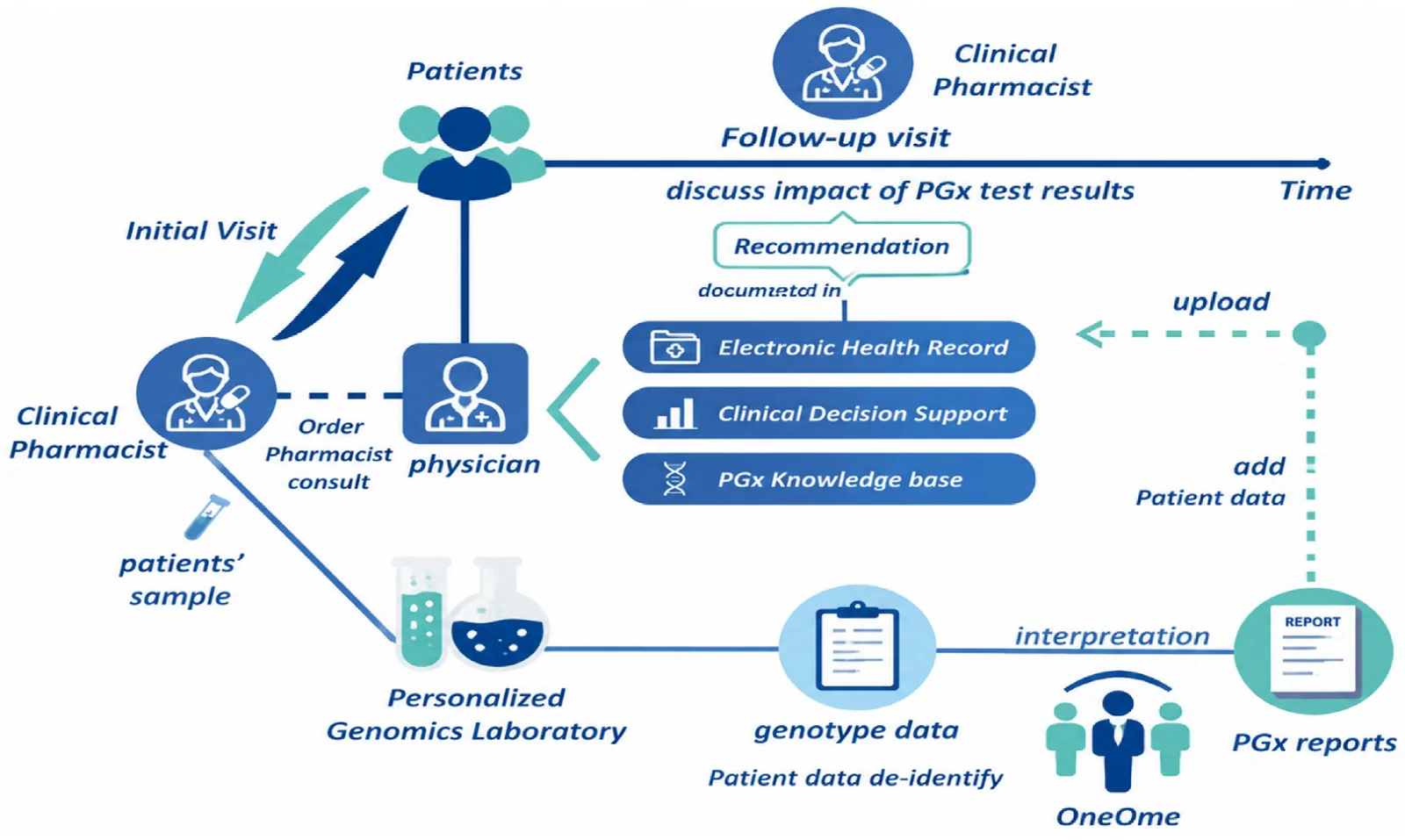
Clinical pharmacist-coordinated pharmacogenomic testing and follow-up workflow. Created by the authors based on the Saudi tertiary care workflow described in [37,38].

Figure 5 illustrates the type of pharmacist-led clinical workflow already emerging in Saudi tertiary centers, in which an initial visit, sample collection, laboratory interpretation and a follow-up visit are coordinated by a clinical pharmacist and physician around a documented pharmacogenomic recommendation.

### 4.2. Governance and Clinical Standardization

There are still no unified national indications in Saudi Arabia to define which patients should be tested, when testing should be done, and which clinical team should be responsible for acting on the result. Differences between international guidelines, formularies and local clinical practice could also add to inconsistent interpretation. Therefore, a national steering committee will be required to endorse Saudi-adapted pathways, allocate clinical responsibilities and keep version-controlled recommendations [12,15]. Institutional innovation structures already operating within the Saudi Ministry of Health, such as dedicated centers that incubate and scale emerging health technologies, could provide a governance template for coordinating PGx pathway approval and national rollout [50].

**Figure 6:**
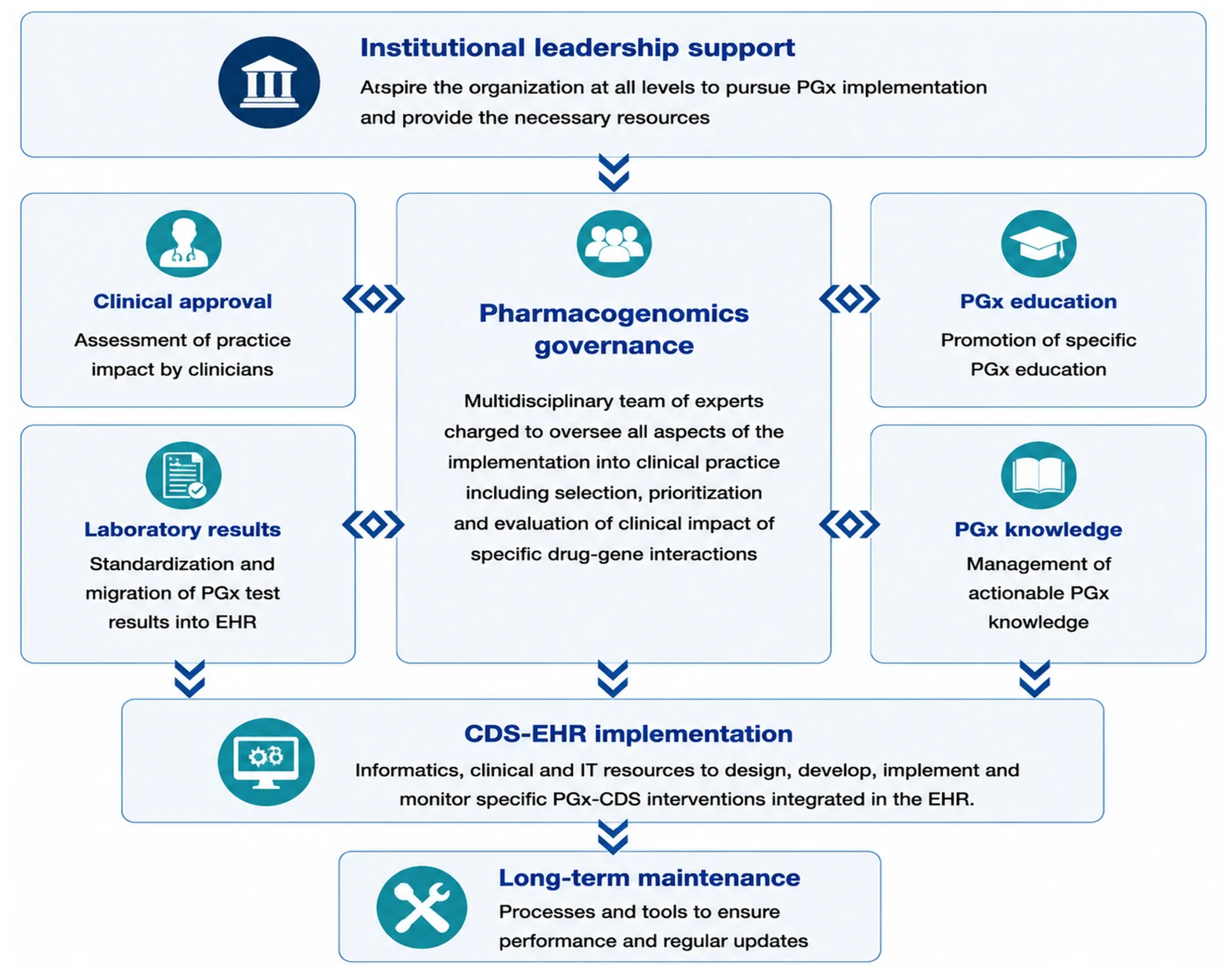
Institutional building blocks for national pharmacogenomics governance. Created by the authors.

Figure 6 summarizes the institutional building blocks that a national PGx governance model would need to coordinate, from leadership sponsorship and clinical approval through laboratory standardization, education, knowledge management and long-term maintenance.

### 4.3. Laboratory and Digital Infrastructure

Availability, allele coverage, turnaround time, and reporting practices could differ between institutions. Consistent analytical performance will require a national reference network, accreditation, and external quality assurance [44]. The PGx results must also be stored as structured, reusable data within the EHR. Poor interoperability, non-computable reports, and poorly designed alerts could contribute to alert fatigue and limit clinical use [45,51]. EHR decision support should therefore be concise, phenotype driven, and linked to a clear prescribing action.

**Figure 7:**
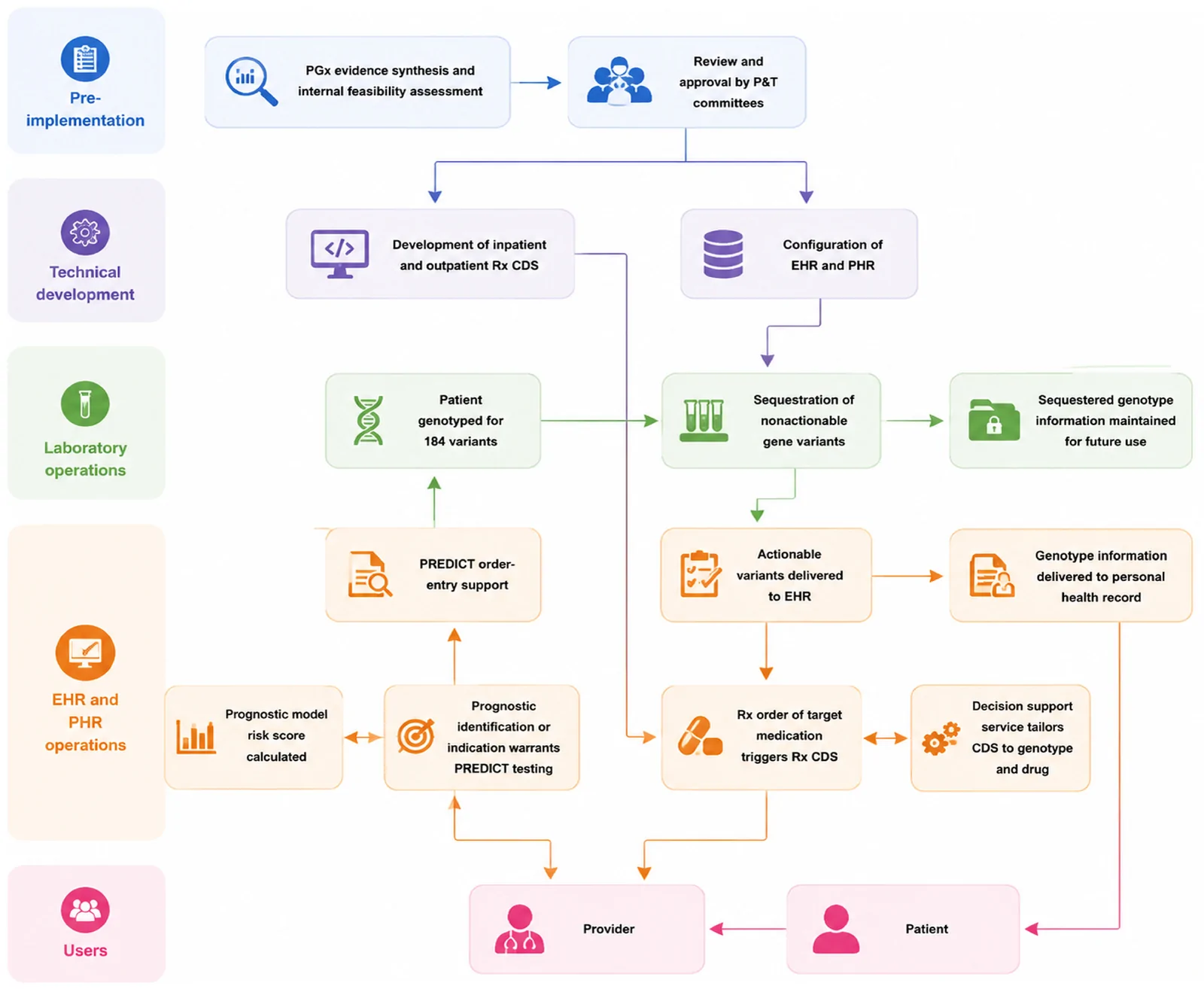
Illustrative pre-emptive pharmacogenomic testing and EHR/PHR integration pipeline. Adapted conceptually from Peterson et al. [52].

Figure 7 shows an illustrative pre-emptive testing pipeline adapted conceptually from the Vanderbilt PREDICT implementation model [52]. It is presented as an implementation example rather than a generic Saudi workflow.

**Figure 8:**
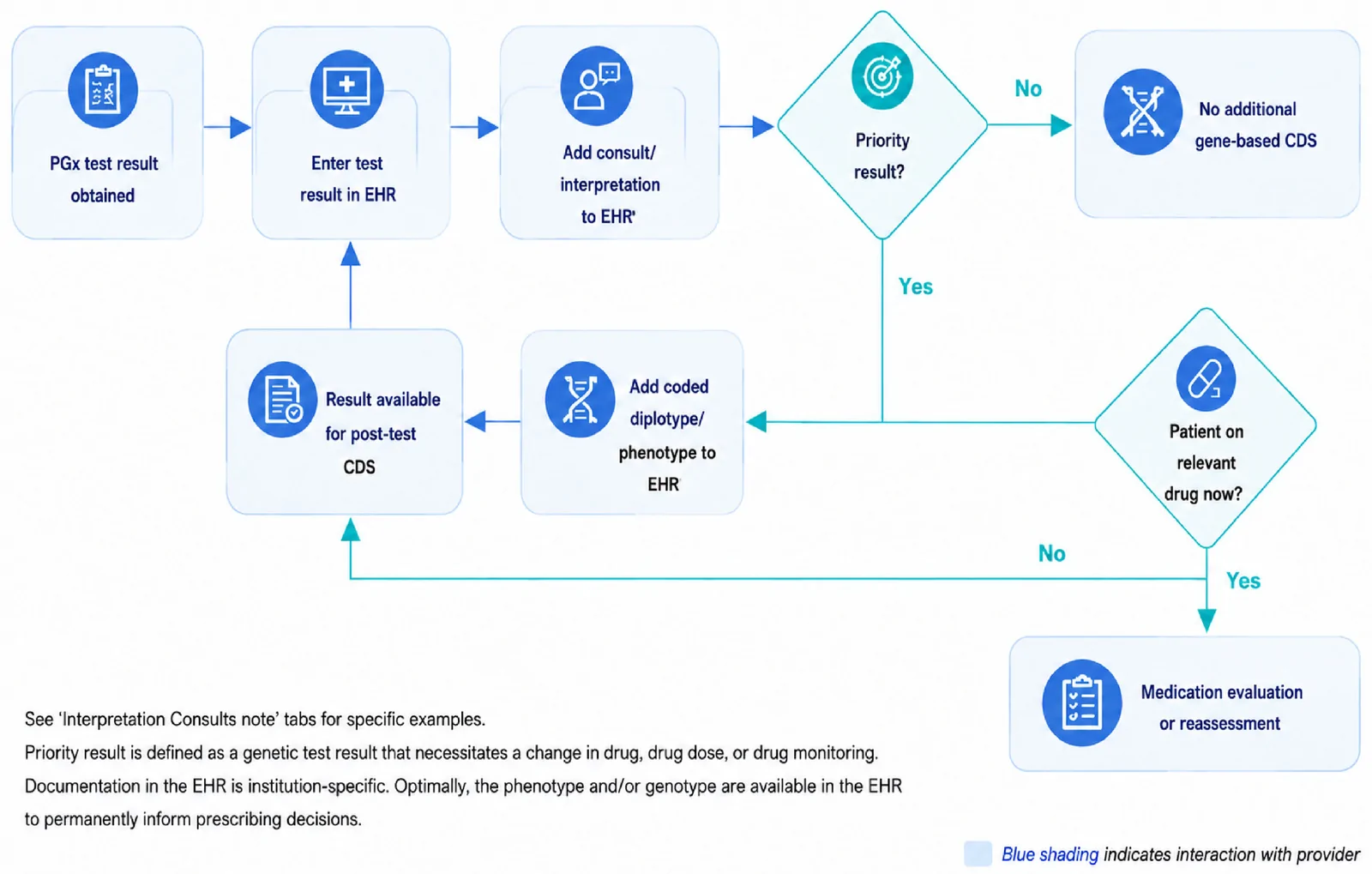
Gene-agnostic clinical decision support implementation logic. Adapted conceptually from CPIC implementation workflows [29,30,32,33,34,35,36].

Figure 8 illustrates gene-agnostic decision logic adapted conceptually from CPIC implementation workflows [29,30,32,33,34,35,36]. It is presented as an illustrative logic model for the proposed clinical knowledge engine (Section 3.5.4).

### 4.4. Workforce Readiness and Clinical Integration

Knowledge and confidence among Saudi healthcare professionals are still inconsistent. Reported barriers include lack of knowledge of test interpretation, prescribing guidelines and clinical workflows [11,12]. Role-specific competencies, practical training and access to specialist consultation will be necessary for successful implementation. Education should come first, before clinical activation, and should continue as recommendations and testing technologies advance. A national survey similarly found that most Saudi physicians and pharmacists reported limited familiarity with pharmacogenomic testing and its clinical application, reinforcing the case for structured, role-specific education before wider clinical activation [53]. Implementation should maintain a multidisciplinary approach and, where feasible, integrate PGx genotyping with relevant phenotyping analyses and careful clinical monitoring. This is particularly relevant to *DPYD*–fluoropyrimidine management and HLA-guided prevention of severe cutaneous adverse drug reactions [54,55].

### 4.5. Reimbursement and Funding

The principal financial requirements include testing, interpretation services, digital infrastructure, and clinical follow-up. The reimbursement pathways are not yet well defined, and economic findings from other countries may not be directly generalizable to Saudi Arabia. Published evaluations suggest that PGx testing can be cost-effective or cost saving, but the value depends on the gene–drug pair, population, testing strategy, and healthcare setting [56]. Local budget-impact and cost-effectiveness analyses should therefore be incorporated into Saudi pilot programs before wider reimbursement decisions are made.

### 4.6. Fair Access and Ethical Governance

PGx implementation raises questions about informed consent, lifelong result storage, secondary use of genomic data, patient access, and potential discrimination. Data processing should comply with the Saudi Personal Data Protection Law and related governance requirements [41]. National implementation must also prevent regional inequality by ensuring access to testing, interpretation, and prescribing support beyond major tertiary centers.

### 4.7. Evidence, Sustainability, and Clinical Uncertainty

Saudi genomic studies provide a strong rationale for PGx implementation, but local prospective evidence regarding clinical effectiveness, cost-effectiveness, regional equity, and long-term sustainability remains limited [7]. Evidence from a small number of tertiary care programs may not be generalizable to all Saudi healthcare settings, where laboratory capacity, digital infrastructure, workforce readiness, and access may differ. Furthermore, targeted panels may not detect rare or structural variants, and imported dosing algorithms may require local validation [8,14]. The readiness ratings and priority tiers should therefore be interpreted as provisional and be validated through the proposed multicenter pilots. Gene–drug priorities should also be periodically reassessed as Saudi allele frequency evidence, prescribing patterns, clinical guidelines, testing capacity, and technologies evolve.

## 5. Recommendations

1. Create a national multidisciplinary PGx steering committee to guide governance, clinical standards, ethical requirements, and implementation priorities.
2. Start with a few high-impact and feasible gene–drug pathways, such as *CYP2C19*–clopidogrel, *DPYD*–fluoropyrimidines, *TPMT*/*NUDT15*–thiopurines, HLA-guided anticonvulsant prescribing, and HLA-B*57:01–abacavir.
3. Conduct multicenter pilot programs in selected Saudi health clusters prior to regional and national scale-up.
4. Build a national accredited laboratory network with standardized testing panels, reporting formats, quality assurance, turnaround times and genotype-to-phenotype interpretation.
5. Store PGx results in electronic health records as structured, portable and reusable data and link them to point-of-care clinical decision support alerts.
6. The Population Health Observatory is proposed as one possible secure platform for data linkage, population-level monitoring, pharmacovigilance, performance evaluation, and ongoing policy improvement.
7. Establish PGx educational and competency programs tailored to the roles of physicians, clinical pharmacists, laboratory professionals, nurses, genetic counselors, and health informatics teams.
8. Develop sustainable reimbursement pathways for testing, interpretation, clinical consultation, and follow-up care supported by Saudi cost-effectiveness and budget-impact studies.
9. Ensure compliance with national privacy laws and introduce clear requirements on consent, data access, secondary use, cybersecurity, non-discrimination and equitable access across regions.
10. Monitor implementation with measurable indicators, such as uptake of testing, time to results, changes in prescribing or adverse drug reactions, clinical outcomes of treatment, cost-effectiveness, decision support overrides, and equity across regions.
11. Expand PGx services only after demonstration of clinical safety and effectiveness, economic value, interoperability, workforce readiness and equitable access through pilot programs.
12. Keep updating a national PGx knowledge system that includes Saudi genomic evidence, real-world clinical results, pharmacovigilance data and updated international guidelines.

## 6. Strengths and Limitations

This framework has several strengths: it integrates Saudi-specific genomic evidence with international implementation experience; links gene–drug prioritization to population health impact and feasibility; and proposes governance, laboratory, digital and clinical components within a single PHO-enabled architecture supported by a phased implementation roadmap.

Several limitations should also be acknowledged. No primary national readiness survey was conducted; the readiness assessment and gene–drug prioritization presented here are informed by published evidence and structured expert judgement rather than a validated quantitative instrument. No national cost-effectiveness dataset was available, so the economic conclusions remain provisional. The clinical outcome evidence for Saudi PGx implementation remains concentrated in a small number of tertiary institutions, some international implementation models may not be directly transferable to the Saudi health system, and the regional variation in readiness may be underestimated. The proposed readiness framework and priority tiers therefore require prospective validation using national prescribing, laboratory, economic, and clinical outcome data generated during the pilot phases described in Section 3.6.

## 7. Conclusions

There is a strong scientific and strategic basis for national implementation of pharmacogenomics in Saudi Arabia. Population-specific genomic evidence, widespread exposure to PGx-relevant medicines, growing laboratory capacity and the ongoing digital health transformation offer a clear opportunity to translate genetic information into safer and more precise prescribing. However, these capabilities are fragmented, and genetic testing alone will not deliver clinical benefit without national governance, standardized laboratory pathways, interoperable EHR data, point-of-care clinical decision support, workforce preparation, reimbursement and appropriate ethical safeguards.

The proposed framework supports a phased implementation starting with high-impact and feasible gene–drug pathways, followed by multicenter pilots, regional expansion and national scale-up. The Population Health Observatory could enable secure data linkage, population-level monitoring and ongoing policy evaluation, with prescribing decisions remaining within clinical systems. Expansion should be informed by demonstrated safety, effectiveness, economic value and equitable access. With synchronized leadership and continuous learning from Saudi real-world evidence, PGx could be a valuable part of the Kingdom’s precision medicine and Vision 2030 healthcare transformation. The priority pathways and PHO-enabled architecture are an evidence-informed implementation proposal rather than a nationally validated model, and national scale-up should remain contingent on the results of the proposed pilot and evaluation stages.

## Figures and Tables

**Table 1:** Global best-practice pharmacogenomics implementation models and implications for Saudi Arabia.

Model/Setting	Model Type	Implementation Approach	Core Components	Main Strength/Evidence	Implication for Saudi Arabia
Netherlands (DPWG)	Guideline development system.	Guideline-integrated prescribing.	Evidence-based dose adjustment, drug avoidance and monitoring recommendations embedded in electronic prescribing and pharmacy systems.	Transforms genotype results into computable prescribing rules at the point of care [18].	Develop Saudi-adapted, machine-readable prescribing rules linked to national EHR and pharmacy systems.
U-PGx/PREPARE	Multicountry implementation program.	Pre-emptive multigene panel implementation.	Twelve-gene panel, standardized phenotype translation, reusable results and decision support across hospitals, primary care and pharmacies.	Demonstrates multicountry feasibility and an approximately 30% reduction in clinically relevant adverse drug reactions among actionable cases [13,19].	Begin with phased panel activation and retain results for lifelong reuse after laboratory and CDS validation.
United States (eMERGE/IGNITE)	Institution-led research and implementation networks.	Institution-led and network-based implementation.	Multidisciplinary teams, local workflow adaptation, EHR integration, clinical decision support and cross-site implementation testing.	Shows that different institutions can implement shared evidence while adapting delivery to local systems [24].	Use multicenter pilots across Saudi health clusters to test interoperability, workflow and scalability before national expansion.
NHS England	National genomic service infrastructure.	Nationally coordinated genomic service model.	National genomic laboratory networks, service commissioning, workforce development and integration of genomic information into clinical records.	Provides a system-level structure for standardization, quality assurance and equitable access [25].	Link PGx to national genomic services, medicine optimization, commissioning and quality standards.
Canada (CPNDS)	Pharmacovigilance and implementation network.	Pharmacovigilance-linked implementation.	Active surveillance of serious adverse drug reactions, genomic analysis, biomarker validation and preventive recommendations.	Connects medication safety surveillance with PGx discovery and targeted prevention [26].	Integrate PGx with national pharmacovigilance and adverse drug reaction reporting systems.
Australia (RCPA)	Professional indication and testing guidance system.	Indication-based testing model.	Testing classified as recommended, consider or no consensus according to evidence, severity, clinical context and service availability.	Clarifies when testing should be ordered, complementing guidelines that explain how results should guide therapy [27].	Create Saudi testing indications, eligibility criteria and reimbursement rules for each approved pathway.
Abu Dhabi (Malaffi)	Regional health information exchange.	Regulator-led health information exchange model.	Population genomic reports made available across participating providers through an emirate-wide health information exchange.	Demonstrates regional portability of PGx reports across healthcare institutions [28].	Use national health information exchange infrastructure so results generated at one institution remain available wherever prescribing occurs.

## Data Availability

No new primary datasets were generated in this study. All information analyzed is available in the sources cited in the article.

## Supplementary Materials

The following supporting information can be downloaded at: https://www.mdpi.com/article/10.3390/genes17091115/s1, Table S1: Search Strategy and Source-Selection Framework; Table S2: Evidence Supporting Readiness Scores [7,8,9,10,12,13,14,15,18,19,22,23,24,25,39,40,41,42,45,56].
